# A study of a culturally focused psychiatric consultation service for Asian American and Latino American primary care patients with depression

**DOI:** 10.1186/1471-244X-11-166

**Published:** 2011-10-13

**Authors:** Nhi-Ha T Trinh, C A Bedoya, Trina E Chang, Katherine Flaherty, Maurizio Fava, Albert Yeung

**Affiliations:** 1 Depression and Clinical Research Program, Massachusetts General Hospital, One Bowdoin Square, sixth floor Boston, MA 02114, USA; 2 Behavioral Medicine Service, Massachusetts General Hospital, One Bowdoin Square, seventh floor Boston, MA 02114, USA; 3 Abt Associates, Inc., 55 Wheeler St # 1A Cambridge, MA 02138, USA

## Abstract

**Background:**

Ethnic minorities with depression are more likely to seek mental health care through primary care providers (PCPs) than mental health specialists. However, both provider and patient-specific challenges exist. PCP-specific challenges include unfamiliarity with depressive symptom profiles in diverse patient populations, limited time to address mental health, and limited referral options for mental health care. Patient-specific challenges include stigma around mental health issues and reluctance to seek mental health treatment. To address these issues, we implemented a multi-component intervention for Asian American and Latino American primary care patients with depression at Massachusetts General Hospital (MGH).

**Methods/Design:**

We propose a randomized controlled trial to evaluate a culturally appropriate intervention to improve the diagnosis and treatment of depression in our target population. Our goals are to facilitate a) primary care providers' ability to provide appropriate, culturally informed care of depression, and b) patients' knowledge of and resources for receiving treatment for depression. Our two-year long intervention targets Asian American and Latino American adult (18 years of age or older) primary care patients at MGH screening positive for symptoms of depression. All eligible patients in the intervention arm of the study who screen positive will be offered a culturally focused psychiatric (CFP) consultation. Patients will meet with a study clinician and receive toolkits that include psychoeducational booklets, worksheets and community resources. Within two weeks of the initial consultation, patients will attend a follow-up visit with the CFP clinicians. Primary outcomes will determine the feasibility and cost associated with implementation of the service, and evaluate patient and provider satisfaction with the CFP service. Exploratory aims will describe the study population at screening, recruitment, and enrollment and identify which variables influenced patient participation in the program.

**Discussion:**

The study involves an innovative yet practical intervention that builds on existing resources and strives to improve quality of care for depression for minorities. Additionally, it complements the current movement in psychiatry to enhance the treatment of depression in primary care settings. If found beneficial, the intervention will serve as a model for care of Asian American and Latino American patients.

**Trial Registration:**

ClinicalTrials.gov: NCT01239407

## Background

The project proposes a randomized controlled trial to evaluate a culturally focused intervention to improve the recognition and treatment of depression in Asian American and Latino American primary care patients at Massachusetts General Hospital (MGH). The intervention entails using multi-lingual consultations and toolkits, for providers and patients, over the course of two visits. Goals of the project are to facilitate a) primary care providers' ability to provide appropriate, culturally informed care of depression in adult Asian American and Latino American patients, and b) patients' knowledge of and resources for receiving treatment for depression.

This intervention focuses on the primary care setting, as ethnic minorities with depression are much more likely to be cared for by primary care physicians (PCPs) than by specialists in mental health [1]. Targeting both the provider and patient in the intervention is important for a variety of reasons. Providers may face challenges in correctly diagnosing depression in patients whose ethnic background is different from their own, due to difficulties in correctly identifying depressive symptoms or using different cues or vocabulary to describe depression [2,3]. In addition, there may be some variation in symptom presentations. For example, Latino Americans may give greater emphasis to somatic complaints than non-Latino Caucasians [4], and as Asian Americans become more acculturated, they tend to report worsening levels of mood symptoms [5]. These varying symptom presentations can differ from what most clinicians are trained to expect, resulting in clinical misdiagnoses [6]. Patients themselves are reluctant to seek treatment; Asian Americans may experience higher levels of perceived stigma related to seeking mental health treatment, particularly from their families [7]. In addition, Latino Americans may have had fewer helpful mental health treatment experiences than non-Latino Caucasians, which may make them less willing to seek treatment in the future [8].

As one solution to bridge this gap between provider knowledge and patient needs, Kirmayer et al. developed the Cultural Consultation Service (CCS) model in 1999 [9,10]. The CCS responded to requests for assistance placed by primary care providers and mental health professionals facing difficulties with the assessment or treatment of ethnically diverse patients. In their evaluation of 100 cases referred to the CCS, Kirmayer's group found that cultural misunderstandings were associated with an increased risk for incomplete assessments, incorrect diagnoses, and inadequate or inappropriate treatment. By providing cultural consultations and formulations based on the DSM-IV cultural formulation model, the CCS effectively improved diagnostic assessment and treatment for their diverse population [9].

In a critical review of the literature, Van Voorhees et al evaluated interventions attempting to reduce disparities between non-Latino Caucasians and ethnic minorities and found that multi-component interventions were successful in improving depressive symptoms and functional status in nearly every study [11]. These interventions utilized a chronic disease management model, complete with case management, enhancement of access to care, and a variety of approaches tailored to the specific system, provider, and patient factors. One study, the "Improving Mood-Promoting Access to Collaborative Treatment" (IMPACT), not only improved all outcomes, but also eliminated ethnic disparities [12]. This trial randomized older adults with depression to usual care or to an intervention including case management, patient education, and either medication or problem-solving psychotherapy within a collaborative care model including available psychiatric consultation. The intervention demonstrated a reduction of depressed mood by at least 50 percent as compared to usual care; however, there was little cultural tailoring of the materials, i.e., the "matching of study intervention goals and materials to the needs and sensitivities of specific populations." In fact, Van Voorhees et al concluded that none of the studies in their review compared culturally tailored interventions with standard interventions in a randomized control trial. They also wondered if "structural changes in the pattern of delivery of mental health services" and "culturally tailored mental health service programs" may prove to be particularly effective to minorities impacted by mental health issues [11]. The proposed intervention seeks to provide such a model to Asian American and Latino American primary care patients - understudied populations in health care disparities intervention research [13,14].

### Preliminary Studies

The first part of the proposed intervention, conducting clinic-wide screening for depression, builds on the work by Dr. Albert Yeung. In one project using widespread depression screening in an Asian American population in primary care in 2004-2005, Yeung and colleagues [15] used a Chinese bilingual version of the Patient Health Questionnaire (CB-PHQ-9) to screen and identify patients with possible depression. Patients who screened positive were interviewed to establish psychiatric diagnosis and to engage them in treatment. Their work demonstrated that depression could be identified in this sample of primary care patients, using a translated screening questionnaire and a culturally sensitive clinical evaluation to engage patients in mental health treatment. In addition, 44% of those who screened positive agreed to come in to be assessed by a psychiatrist; among those who were then diagnosed with MDD, 93% were willing to accept treatment. These findings demonstrate that screening minority patients in primary care settings can be both a feasible and effective tool to identify patients likely to have clinical depression.

Mischoulon et al. reported that screening in primary care settings may not be enough; independent screening by psychiatrists in primary care settings may not be adequate to ensure appropriate management of depression by PCPs [16]. In this study, patients were screened for MDD in a community-based primary care health center. For those who met criteria for MDD, a letter was mailed to their PCP informing them of their patient's diagnosis of MDD. Forty outpatients, of whom 29 (72 percent) were Latino American, were found to meet criteria for MDD. Medical record charts were reviewed 3 months later to determine the PCPs' management following the diagnosis. Of the 38 patients who remained in the study at 3 months, 20 (53 percent) received no intervention for depression from the PCP, and of these, 14 (70 percent) were Latino American. Only five (13 percent) were prescribed an antidepressant by their PCP, nine (24 percent) were referred to mental health services for medication, psychotherapy or combination treatment, and four (11 percent) were prescribed an antidepressant and then referred to mental health services. Mischoulon and colleagues concluded that possible explanations for this lack of treatment of depression may include time constraints during primary care visits, patient and/or physician reticence, and insufficient education of PCPs about clinical depression. These findings suggest that screening and notification must be supplemented by additional interventions if they are to have an impact on the care of depression.

The second part of this proposed intervention derives from a pilot project from our group that applied a culturally focused psychiatric consultation service for a group of high service-utilization patients. In the initial pilot, the service was based on the Cultural Consultation Service (CCS) model developed by Kirmayer et al [9,10]. As part of the consultation service, members of the project team met monthly with case managers who were following a group of chronically, medically ill patients. Cases with difficult psychiatric issues were discussed, and formal consults could be requested for patients whose presentation and treatment was complicated by cultural issues. Members of the consultation service also met regularly to discuss cases. Results of the initial needs assessment indicated that case managers perceived a strong need for such a culturally focused psychiatric consultation service, but tended to refer patients for psychosocial issues rather than for psychiatric diagnostic assessment. Similarly, in a six-month follow-up to the culturally focused psychiatric clinical intervention, case managers were asked about their overall perception of the utility of the service and obstacles to its use. Based on their responses, case managers perceived a significant value, but tended to underutilize the service. This experience suggested four hypotheses. The consultation service would benefit from: a) focusing on a specific psychiatric diagnosis; b) targeting patients at their primary care providers' offices directly, providing a greater ability to connect with minority patients than an intervention that serves as a liaison to staff; c) making the consultation service more user-friendly; and d) enriching the intervention with patient-focused materials, in order to maximize patient knowledge and skills. These findings further supported the recruitment of patients through screening for those who meet criteria for likely depression, rather than relying solely on referrals by PCPs.

A final component of the CFP intervention was based on the team's experience with adapting cognitive behavioral interventions for the treatment of depressive disorders in chronically ill Latino Americans [17,18]. For example, in a sample of HIV-positive foreign-born Latino Americans, a cognitive behavioral intervention was linguistically/culturally adapted and administered by bilingual/bicultural staff. Participants received either a 10-week cognitive behavioral stress management (CBSM) intervention or a one-day seminar. Results indicated that, compared to those who only received the seminar, participants who received the CBSM intervention reported significantly less anger, maladaptive coping, and HIV-related symptoms [19]. These results led to recommendations for including culturally-adapted cognitive behavioral therapy techniques within clinical interventions for chronically-ill Latinos, for example, by addressing cultural factors such as familismo and respeto.

### Study Aims

**Figure 1:**
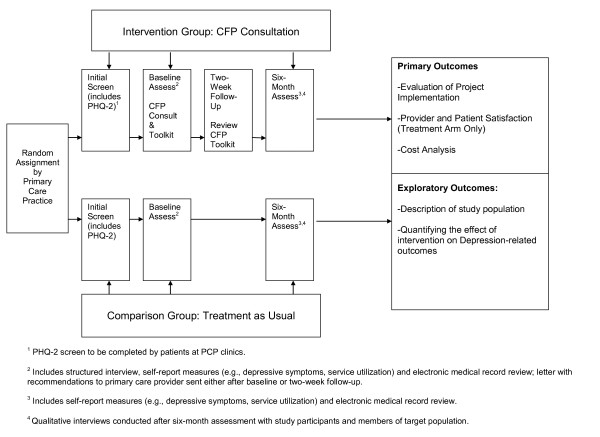
**Clinical Research Intervention Design**.

This study is a randomized controlled trial of a culturally focused psychiatric consultation model for Asian American and Latino American primary care patients with depression (see Figure 1). The *primary aim *of the study is to determine the feasibility, satisfaction and cost associated with implementing a CFP consultation service within a primary care setting at MGH. Additionally, an *exploratory aim *serves to describe (e.g., demographic factors; level of depression; service utilization) the participant patient population at screening, recruitment and enrollment, as well as to determine which, if any, of these variables influence participation in the study (e.g., consent to be contacted).

## Methods/Design

### Overview

This is a randomized controlled trial of the CFP intervention. During the study period, all patients in participating primary care clinics will receive a screening form for symptoms of depression; data on age, gender, race, ethnicity and primary language will be obtained at the time of screening. Asian American and Latino American patients who screen positive for clinical depression will be contacted by study staff and invited to participate in the CFP study. Eligible patients will be randomized either to the intervention arm or usual care based on their primary care provider. Patients in the intervention arm will receive the CFP intervention over two visits and then called at six-months by study staff to administer follow-up questionnaires (described below). Patients in the usual care arm will be called at baseline and at six-month follow-up by study staff to administer questionnaires (described below). To evaluate the cultural components of the study, a number of qualitative interviews will also be conducted with participants in the intervention and usual care arms, and with members of the target population who did not participate in the study. Please see Figure 1 for the study flowchart.

### Description of Intervention

The CFP intervention includes two major components:

1) Patients receiving the intervention will undergo a CFP consultation assessment by a member of a multidisciplinary team of mental health providers (e.g., psychiatrists, psychologists) who are bilingual and/or bicultural, trained in culturally competent techniques, and familiar with the cultures and languages of the clients served. The Engagement Interview Protocol and DSM-IV-TR Cultural Formulation model will be utilized to make a culturally appropriate diagnosis, assess the patient's psychiatric needs in a cultural context, conduct a culturally focused intervention, engage the patient, and make recommendations to the PCP [15,20,21]. The CFP clinician will work collaboratively with the patient's PCP to communicate diagnostic and treatment recommendations after the two-week follow-up visit. Given the critical nature of communicating the CFP findings to the PCP, the CFP clinician will work with the PCP to determine his preferences for communication. Recommendations can be given to the PCP by multiple avenues: by e-mailed letter, and/or by page or phone call as needed. Clinicians will make themselves available to PCPs on an as-needed basis throughout the study period.

2) Patients will receive a culturally appropriate CFP consultation patient toolkit, available in their language of preference (i.e., English, Spanish, Chinese, or Vietnamese), as well as training in using the toolkit materials. The patient toolkit was added to enrich the intervention by providing patients with psychoeducation, handouts on managing depression based on cognitive behavioral therapy techniques, and information on community resources [22]. To help in the management of their depression, patients will be encouraged to complete these materials and bring them to visits with their PCP. In addition, within two weeks of the initial consultation, patients will attend a follow-up visit with the CFP clinician to review and answer their questions about the treatment of their depressive symptoms. The CFP clinician will also review patients' understanding of the toolkit, including their use of the cognitive-behavioral based handouts. To add flexibility in follow-up for patients, these visits can be done in person or over the phone.

### CFP Intervention Training

At the beginning of the study, CFP clinicians attend four hour-long training sessions on the CFP consultation study procedures to ensure that the intervention is applied in a standard fashion. Clinicians will audiotape all CFP sessions, and a weekly group supervision meeting for CFP clinicians will be held to review cases and provide supervision for issues related to clinical diagnosis and intervention. Finally, the CFP consultation service will meet weekly to discuss consultation requests, follow up on the impact of consultations, and to address other logistic issues.

### Inclusion/Exclusion Criteria

Patients will be included based on the following criteria: adults who 1) are 18 years of age or older; 2) are members of either targeted Asian American or Latino American minority groups; 3) screen positive for symptoms of depression; and 4) are able to consent to study participation. Patients will be excluded if they have active unstable, untreated psychiatric illness precluding participation in the study (e.g., actively suicidal or homicidal or actively psychotic). Patients in the intervention arm will be excluded if they have bipolar disorder.

### Target PCP practices

The intervention will be conducted at four MGH primary care practices; each PCP practice consists of multiple sub-practices called pods.

### Recruitment strategy

All patients in participating primary care clinics will be offered screening for the symptoms of depression by the Patient Health Questionnaire-2 (PHQ-2) as part of their routine primary care visit [23]; data on age, gender, race, ethnicity and primary language will be obtained at the time of screening. Asian American and Latino American patients who screen positive for clinical depression will be called by study staff and invited to participate in the CFP consultation intervention. Patients also may be referred by participating PCPs. For the qualitative interviews, a subset of study participants will be invited to participate after the six-month follow-up visit, as well as eligible members of the target population who not participate in the study.

### Randomization scheme

Primary care patients at MGH will be randomized by pods, which comprise multiple clusters of 5-6 PCPs within each practice site. Using electronic medical records data from 2007, practices will be matched based on an index composed of two variables: 1) the number of patients who are members of the target minority groups and 2) the rate of diagnosed depression. Within each pair, one practice will be randomized to participate in the intervention and the other will be assigned to the "usual care" arm.

### Target Health Condition

All patients in the intervention and usual care (control) arms will be screened for clinical depression using the self-rated PHQ-2 at their primary care visit. Both patients and PCPs will be notified of positive screening results for all patients assessed. For the intervention arm, diagnoses of specific depressive disorders (major depressive disorder, dysthymia, depressive disorder NOS, adjustment disorder with depressed mood) will be established during the baseline clinical interview using DSM-IV-TR criteria [21].

### Definition of Usual Care

PCPs of those patients who screen positive on the PHQ-2 in the usual care arm will receive written feedback on their patients' PHQ-2 results, in addition to a standard list of referrals to MGH mental health resources. At the end of the study, patients in the usual care arm will be offered the intervention.

### Human Subjects Approval

All study procedures have been approved by the hospital Institutional Review Board (IRB).

### Analytic Plan

*Description of Measures *(See Table 1):

**Table 1:** Patient measures administered over study period.

PATIENT MEASURES BY TIMEPOINT					
	**Screen**	**Baseline Assessment**	**2 Week Follow-Up^+^**	**6 Month Follow-Up^+^**	**Qualitative Interview^+^**

MINI International Neuropsychiatric Interview (MINI)*		X			

Quick Inventory of Depressive Symptomatology (QIDS-SR)		X		X	

Resource Utilization Questionnaire		X		X	

CFP Consultation, Assessment & Toolkit*		X	X		

Global Assessment of Functioning (GAF)*		X			

Patient Health Questionnaire (PHQ-2)	X				

Patient Satisfaction Questionnaire				X	

Schwartz Outcome Scale (SOS-10)		X	X	X	

Demographic Questionnaire		X			

Qualitative interview					X

*Intervention arm only.

^+ ^Visit may be conducted by phone.

### Initial Screen

On the screening form, patients will be administered the PHQ-2 and asked additional questions about gender, age, race, ethnicity, and language spoken at home. Using a cutoff score of two or above (out of a possible total score of six), the PHQ-2 has been validated for diagnosing any clinical depressive disorder with a 82.1 percent sensitivity, 80.4 percent specificity, and a positive predictive value of 48.3 percent (assuming 18 percent prevalence of any depressive disorder) [23]; it consists of the first two questions of the PHQ-9, which has been translated into Chinese, Vietnamese, and Spanish [23-27]. The screening form will be available in multiple languages (English, Spanish, Chinese, and Vietnamese).

### Baseline Visit

Both patients in the intervention and usual care arm will give consent to participate in the study at the beginning of the baseline visit. In the intervention arm, assessment measures used at baseline to characterize the intervention patient population will include the Mini International Neuropsychiatric Interview (MINI) [28], Quick Inventory of Depressive Symptomatology-Self Rated Scale (QIDS-SR) (see Appendix 2) [29], Global Assessment of Functioning (GAF) [21], and Schwartz Outcome Scale (SOS-10) [30]. Questionnaires regarding the patients' demographics and utilization of resources will also be assessed. In the usual care arm, for target minorities who screen positive, the assessment measure will include the QIDS-SR and SOS-10.

### Six-month follow-up

Patients will have the option to complete the visit over the phone or in person. In both the intervention and usual care arms, patients will be given the QIDS-SR self-rated questionnaire and asked a brief "Resource Utilization Questionnaire" with questions regarding use of antidepressant medications, referrals/visits to mental health specialists, or alternative providers both in and out of the MGH system. The electronic medical record will also be reviewed at baseline and at the six-month follow-up for both intervention and usual care arms to obtain depression diagnoses, prescription of antidepressant medications, and referrals/visits to mental health specialists in the system.

### Sample size and power

Initially, we planned for a total sample size of 506 patients, split evenly between the intervention and usual care groups. We calculated this sample size to yield 84 percent power to detect a difference in the reduction of depressive symptoms on the QIDS-SR by 50 percent in the intervention arm versus 35 percent in the treatment arm, paralleling the experience of patients in the intervention arm in STAR*D, an multi-center effectiveness study of Major Depression in primary care settings [31] as well as the experience of patients in usual care arm in several studies [32-34]. However, given the multiple recruitment challenges we have faced to date, we have revised our total sample size to 120 patients, split evenly between intervention and usual care groups. Although this sample size is significantly smaller, the results of both the qualitative and exploratory analyses of this study will inform further iterations of this intervention and study design.

### Qualitative analysis

The project's implementation process and satisfaction (providers' and patients') with the CFP consultation intervention will be assessed in several ways. Qualitative data will be collected through interviews, meetings and conversations with the project and primary care practice staff, as well as reviews of project records and meeting summaries. Interviews will be transcribed and translated, with content analysis used to assess for convergent and divergent themes.

The patient interviews will include samples of Asian American and Latino American patients. In addition, interviews will be conducted with members of the target population who did not participate in the study. The interviews will be conducted by telephone, or in person as needed. The interviews will assess the extent of patients' involvement with the project or perception of this kind of intervention. All participants will be asked for their opinions about offering this service through their primary care office and the screening process. In addition, intervention participants will be surveyed about satisfaction, use of the toolkit, opinions about the cultural tailoring of the intervention, and asked for their suggested changes to the intervention. Intervention patients will also be asked about their follow-up on recommendations from the consultation and discussions - if any - with their PCP resulting from the intervention. Usual care participants will be asked about their use of mental health services, their understanding of the study when they enrolled, and their views on what they believe would be important to include within toolkits or a culturally focused psychiatric consultation service.

Using open- and closed- ended questions, the project staff and practice staff (including PCPs, practice managers and frontline staff) will be asked about their perceptions of, experiences with, and satisfaction with the project's implementation, including the screening and referral process. The organizational structure and resources of each participating primary care practice will be characterized at the beginning of the project to assist in the understanding and interpretation of implementation differences across the sites. Through the review of organizational data and regularly collected project data and the interviews, the identified organizational issues will be addressed with the practices, possibly identifying the need for procedural changes or additional staff training.

Additionally, the costs of implementing and continuing the program will be assessed. Initial startup costs and the ongoing costs of the intervention will be tracked. Both the direct dollar outlays associated with program implementation and the dollar values of program resources not reflected in the program budget will be calculated. While the project budget and expenses can be calculated directly from project data, work will be done with practices to estimate the cost of consultations and coordinating care.

Other questions to be asked within this cost analysis will be whether patients' utilization of practice and hospital services--including primary care, emergency department and inpatient visits and their associated costs--changed as a result of participating in this project. Costs of the care provided to the patients will also be compared to the cost of usual care in the control group. Using the figures above, the total range and average costs of care for the patients receiving the intervention will be calculated. A preliminary analysis also will be undertaken to look at differences in the use of services in the treatment versus the control group, i.e., if the two groups differ in their utilization and costs of primary care, emergency department visits and inpatient care. Such cost analyses may provide preliminary estimates of the costs of implementing such interventions in academic medical centers.

### Exploratory analysis

Descriptive analyses will be performed to provide information related to the study's experience conducting the initial screening patients within their primary care clinics.

Data from initial screens will be utilized to provide a greater description of the patient population at screening, recruitment and enrollment (e.g., race; ethnicity; preferred language; age; gender; level of depression (i.e., PHQ-2 score); service utilization, as well as identify which of these variables, if any, served to influence participation in the study. This information will allow us to describe, for example, whether consent to be contacted differs by level of depression and whether this is moderated by demographic factors (e.g., race, ethnicity, gender). Additionally we will provide additional data describing rates of enrollment as compared to the total screened.

Secondary exploratory analyses will explore the impact of the intervention on depression. Patients' scores on the QIDS-SR will be compared between the usual care and intervention arms. To measure changes in racial and ethnic disparities in depression recognition and treatment, rates of depression diagnoses will be compared from baseline to six-month follow-up for the intervention and usual care arms. As measures of the use of any ongoing specialty mental health care, the rates of prescription of pharmacotherapy and referrals/visits to mental health specialists as provided by patient self-report will be compared at baseline and at the six-month follow-up. Data analysis will be conducted using STATA statistical software [35].

A general linear model that accounts for clustering at the PCP level will be used. Analyses of the main outcomes will include as covariates any patient or physician factors that differed significantly between study arms. Outcomes regarding diagnosis and treatment will be assessed using general estimating equations with a binomial link and standard errors will be adjusted to account for clustering within practice. Separate analyses will be conducted for each outcome (i.e., dependent variables) -- changes in depressive symptoms, diagnosis of and treatment (prescription of antidepressant medications; referrals/visits to mental health specialists) for depression at six months after enrollment. The effect of study arm on each outcome will be assessed, controlling for significant differences emerging from the bivariate analyses.

## Discussion

The intervention is designed to provide enhanced recognition and treatment of depression in Asian American and Latino American primary care patients, while placing minimal additional burden on the existing primary care system at MGH. Several challenges to the implementation of this intervention exist, including minimizing burden to participating sites, recruitment and retention issues, and language and literacy issues.

Regarding the first challenge, obtaining staff and partnership buy-in during the grant-writing stage, and soliciting feedback from all levels of administration during every step of the process of designing and implementing the study has been instrumental. Regular meetings with participating sites are crucial to clarify workflow, questions about the service, and other issues that may arise. Keeping the protocols simple and easy to implement will be critical.

As with any clinical research intervention, recruitment and retention (e.g., dropout, loss to follow-up) are concerns (40). This is particularly relevant for recruitment and retention of minorities. Within recruitment and retention, the CFP team will consider how and which cultural issues are applicable in order to target Asian American and Latino American primary care patients. Among these groups, examples of such characteristics may include language and literacy, use of complementary and alternative treatments, the perceived importance of the quality of interpersonal relationships between the patient and CFP team, and the relationship between the PCP practices and the CFP team.

To address language and literacy issues, all materials (e.g., consent forms, study instructions) and the CFP consultations will be provided in the patient's preferred primary language, and materials for the toolkit will be developed at a sixth-grade reading level. The patient toolkit has also been prepared in audio CD format for patients with literacy issues. Recruiting research assistants and clinical staff with Spanish, Mandarin, Cantonese and Vietnamese proficiency are crucial to the team.

If patients and providers indicate strong satisfaction with the CFP intervention, it is hoped that the intervention can be expanded through a large scale randomized controlled trial of the intervention. Findings from such a study will provide evidence for the effectiveness of this model to improve the mental health of minorities in primary care settings.

Although this intervention is being piloted at MGH with Asian American and Latino American patients, the goal is ultimately to make it more generalizable with the ability to disseminate the intervention to different settings. To this end, a crucial end-product of this intervention will be the creation of a manualized "start-up guide" which will include the tools used to develop the CFP consultation, including conceptual models, protocols used for assessment, implementation process and its evaluation. A detailed evaluation will be conducted to understand the implementation process and costs (e.g., start-up and implementation). This implementation evaluation and cost analysis will inform the generalizability of this intervention. Organizations seeking to implement the CFP consultation intervention could use this guide to inform the development of an intervention appropriate to their setting.

Regarding dissemination, this consultation model could be used in any health care setting with outpatient clinics and sufficient staffing capacity to create a psychiatrically focused consultation service. Certainly, questions arise regarding the ability of an organization to implement this intervention with a significant minority population but few bicultural/bilingual providers, or in a setting where providers have not been trained to conduct similar culturally focused interventions. Such an organization may need concrete details regarding intervention implementation as discussed above. Similarly, the organization may benefit from seeking outside supervision and support from others experienced in conducting culturally focused interventions. However, such supervision and support is available and more readily accessible. One of the senior members of our team, Dr. Yeung, is an expert in the field of telepsychiatry and is currently developing a model of telepsychiatry consultation via the internet for Chinese American immigrants with depression. He is interested in eventually expanding a network of multicultural clinicians that can serve as consultants to patients in areas with underserved minorities, or as consultants to organizations planning to implement this model. The MGH CFP consultation team would be eager to serve in this capacity. If this intervention is disseminated to other organizations, further research regarding the generalizability and dissemination process of this intervention would inform this approach.

By improving the identification and treatment of depression in Latino American and Asian American populations, this intervention will help reduce the well-known problems with under-recognition and under-treatment of these minority patient groups. This CFP consultation service complements the current movement in psychiatry to enhance the treatment of depression in primary care settings, especially useful for minority patients who are unwilling or unable to access specialty mental health care.

## Conclusion

The proposed CFP intervention complements the current movement in psychiatry to enhance the treatment of depression in primary care settings. This project serves as a model for understanding both a) the effectiveness of improving the mental health care of minorities in primary care settings; and b) the feasibility of this intervention through the use of an implementation evaluation and cost analysis.

## Competing interests

Except for Dr. Fava, whose financial competing interests are listed below, all other contributors report they have no competing interests.

Maurizio Fava, MD - Disclosures (updated May 31, 2011)

Lifetime Research Support:

Abbott Laboratories; Alkermes, Inc.; Aspect Medical Systems; AstraZeneca; BioResearch; BrainCells Inc.; Bristol-Myers Squibb; Cephalon, Inc.; CeNeRx BioPharma; Clinical Trials Solutions, LLC; Clintara, LLC; Covidien; Eli Lilly and Company; EnVivo Pharmaceuticals, Inc.; Euthymics Bioscience, Inc.; Forest Pharmaceuticals, Inc.; Ganeden Biotech, Inc.; GlaxoSmithKline; Icon Clinical Research; i3 Innovus; Johnson & Johnson Pharmaceutical Research & Development; Lichtwer Pharma GmbH; Lorex Pharmaceuticals; NARSAD; NCCAM; NIDA; NIMH; Novartis AG; Organon Pharmaceuticals; PamLab, LLC.; Pfizer Inc.; Pharmavite^® ^LLC; Photothera; Roche; RCT Logic, LLC; Sanofi-Aventis US LLC; Shire; Solvay Pharmaceuticals, Inc.; Synthelabo; Wyeth-Ayerst Laboratories

Advisory/Consulting: financial remuneration is $0 unless otherwise noted

Abbott Laboratories; Affectis Pharmaceuticals AG; Alkermes, Inc.; Amarin Pharma Inc.; Aspect Medical Systems; AstraZeneca; Auspex Pharmaceuticals; Bayer AG; Best Practice Project Management, Inc.; BioMarin Pharmaceuticals, Inc.; Biovail Corporation; BrainCells Inc; Bristol-Myers Squibb; CeNeRx BioPharma; Cephalon, Inc.; Clinical Trials Solutions, LLC; CNS Response, Inc.; Compellis Pharmaceuticals; Cypress Pharmaceutical, Inc.; DiagnoSearch Life Sciences (P) Ltd.; Dinippon Sumitomo Pharma Co. Inc.; Dov Pharmaceuticals, Inc.; Edgemont Pharmaceuticals, Inc.; Eisai Inc.(2010-5500.); Eli Lilly and Company; ePharmaSolutions; EPIX Pharmaceuticals, Inc.; Euthymics Bioscience, Inc.; Fabre-Kramer Pharmaceuticals, Inc.; Forest Pharmaceuticals, Inc.; GenOmind, LLC; GlaxoSmithKline; Grunenthal GmbH; i3 Innovus; Janssen Pharmaceutica; Jazz Pharmaceuticals, Inc.; Johnson & Johnson Pharmaceutical Research & Development, LLC (2010-$1500; 2010-$4000.); Knoll Pharmaceuticals Corp.; Labopharm Inc.; Lorex Pharmaceuticals; Lundbeck Inc.; MedAvante, Inc.; Merck & Co., Inc.; MSI Methylation Sciences, Inc.; Naurex, Inc.; Neuronetics, Inc.; Novartis AG; Nutrition 21; Orexigen Therapeutics, Inc.; Organon Pharmaceuticals; Otsuka Pharmaceuticals; PamLab, LLC.; Pfizer Inc.(2011-$3500.); PharmaStar; Pharmavite^® ^LLC.; PharmoRx Therapeutics; Precision Human Biolaboratory; Prexa Pharmaceuticals, Inc.; Puretech Ventures; PsychoGenics; Psylin Neurosciences, Inc.; Rexahn Pharmaceuticals, Inc.; Ridge Diagnostics, Inc.; Roche; RCT Logic, LLC; Sanofi-Aventis US LLC.; Sepracor Inc.; Servier Laboratories; Schering-Plough Corporation; Solvay Pharmaceuticals, Inc.; Somaxon Pharmaceuticals, Inc.; Somerset Pharmaceuticals, Inc.; Sunovion Pharmaceuticals; Synthelabo; Takeda Pharmaceutical Company Limited; Tetragenex Pharmaceuticals, Inc.; TransForm Pharmaceuticals, Inc.; Transcept Pharmaceuticals, Inc.; Vanda Pharmaceuticals, Inc.

Speaking/Publishing: financial remuneration is $0 unless otherwise noted

Adamed, Co; Advanced Meeting Partners; American Psychiatric Association; American Society of Clinical Psychopharmacology; AstraZeneca; Belvoir Media Group for editing a newsletter (2008-$12, 000.; 2009-$12, 000.; 2010-$12, 000.; 2011-$3000.); Boehringer Ingelheim GmbH; Bristol-Myers Squibb; Cephalon, Inc.; CME Institute/Physicians Postgraduate Press, Inc. for editing supplements & CME web activity (2008-$5500.; 2009-$8500.; 2010-$750.; $3500. 2011); Eli Lilly and Company; Forest Pharmaceuticals, Inc.; GlaxoSmithKline; Imedex, LLC; MGH Psychiatry Academy/Primedia; MGH Psychiatry Academy/Reed Elsevier for speaking at symposium (2008- $13, 000.; 2009-$7800.; 2010-$6535.);MGH Psychiatry Academy for speaking at symposium 3/26/11 (2011-$2500.) Novartis AG; Organon Pharmaceuticals; Pfizer Inc.; PharmaStar; United BioSource, Corp.; Wyeth-Ayerst Laboratories

Equity Holdings: Compellis

Royalty/patent, other income: Patent for SPCD and patent application for a combination of azapirones and bupropion in MDD, copyright royalties for the MGH CPFQ, SFI, ATRQ, DESS, and SAFER. Patent for research and licensing of SPCD with RCT Logic. Royalty from Lippincott, Williams & Wilkins for Handbook of Psychiatric Drug Therapy (2010-$835.) Royalty from Wolters Kluwer Health Inc., (2010: $2954. for 2009; 2011-$1599. for 2010)) World Scientific Publishing Co. Pte.Ltd. (2011: $544. for 2010)

## Authors' contributions

NT and CAB drafted this paper which was added to and modified by all other authors. NT, CAB, TC, AY developed the content of the CFP consultation service; NT, CAB, TC, AY, MF, and KF contributed to the design of the study and analytic strategy. All authors have read and approved the final manuscript.

## Pre-publication history

The pre-publication history for this paper can be accessed here:

http://www.biomedcentral.com/1471-244X/11/166/prepub
